# Cryptogenic chronic hepatitis: looking for an ideal diagnostic algorithm

**DOI:** 10.3389/fgstr.2023.1209000

**Published:** 2023-08-01

**Authors:** Guilherme Grossi Lopes Cançado, Aline Coelho Rocha Candolo, Mateus Jorge Nardelli, Patricia Momoyo Zitelli, Daniel Ferraz de Campos Mazo, Claudia Pinto Oliveira, Marlone Cunha-Silva, Raquel Dias Greca, Roberta Chaves Araújo, Amanda Sacha Paulino Tolentino Alustau, Cláudia Alves Couto, Gabriel Rezende de Lima Roque, Alberto Queiroz Farias, Flair José Carrilho, Mário Guimarães Pessôa

**Affiliations:** ^1^ Instituto Alfa de Gastroenterologia, Hospital das Clínicas, Universidade Federal de Minas Gerais, Belo Horizonte, Brazil; ^2^ Division of Clinical Gastroenterology and Hepatology, Department of Gastroenterology, Hospital das Clínicas, Universidade de São Paulo, São Paulo, Brazil; ^3^ Division of Gastroenterology (Gastrocentro), School of Medical Sciences, Universidade Estadual de Campinas, Campinas, Brazil; ^4^ Gastroenterology Division, Ribeirão Preto Medical School, Universidade de São Paulo - Ribeirão Preto, Ribeirão Preto, Brazil

**Keywords:** cryptogenic hepatitis, cryptogenic cirrhosis, metabolic associated fatty liver disease, non-alcoholic fatty liver disease, diagnosis, algorithm

## Abstract

**Introduction:**

Cryptogenic chronic hepatitis is a growing cause of liver transplants, affecting 5%–15% of patients with chronic liver diseases. This study aimed to identify underlying causes of cryptogenic liver disease in a Brazilian cohort and propose a new diagnostic algorithm, including investigation for metabolic-dysfunction-associated fatty liver disease (MAFLD) and lysosomal acid lipase deficiency (LAL-D).

**Methods:**

A retrospective analysis was conducted on 326 patients with presumed cryptogenic hepatitis.

**Results:**

Using Czaja’s algorithm, non-alcoholic fatty liver disease was diagnosed in 21.3% of patients, while alpha-1 antitrypsin deficiency, alcoholic liver disease, autoimmune hepatitis, hemochromatosis, biliary-related hepatitis, viral hepatitis, Budd–Chiari syndrome, glycogenosis, drug-induced liver injury, and Wilson’s disease were diagnosed in smaller proportions (< 3.5% each). LAL-D was found in 1% of patients, and 53.6% of patients remained with cryptogenic hepatitis. The etiology of the liver disease in a subset of patients undergoing liver transplantation was updated *post hoc* based on explant histology, and non-alcoholic steatohepatitis was found in 52.5% of patients. By incorporating the concept of MAFLD, the new algorithm could diagnose 49.1% of patients, reducing the number of individuals without an etiological diagnosis by 11.4%.

**Conclusion:**

One-third of patients with initially presumed cryptogenic liver disease were diagnosed with MAFLD. LAL-D should be considered in patients with chronic liver disease of unknown etiology. The updated diagnostic algorithm proposed in this study could improve diagnostic accuracy and aid in the management of patients with cryptogenic hepatitis.

## 1 Introduction

Cryptogenic chronic hepatitis is a persistent inflammation of the liver that is unexplained by comprehensive clinical, laboratory, and histological investigations (1). It affects 5%–15% of patients with chronic liver disease (2). Cryptogenic cirrhosis is diagnosed in 5%–30% of patients with cirrhosis, and it is found in 3%–14% of adults awaiting liver transplantation (3–6). The diagnosis depends on the diligence, expertise, and rigor of the examiner, as well as on the laboratorial and anatomopathological resources available for a comprehensive investigation.

It is also important to mention that cryptogenic hepatitis may reflect a late phase of disease in which classical findings have been lost or obscured transiently (1). On the other hand, the new definition of metabolic-associated fatty liver disease has recently increased the potential for the etiological diagnosis of several patients previously labeled with cryptogenic hepatitis, especially among cirrhotics (7–10). Lysosomal acid lipase deficiency (LAL-D), silent autoimmune hepatitis, occult viral hepatitis, alcohol-related liver injury, and alpha-1 antitrypsin deficiency have also been linked to the cryptogenic liver disease spectrum and can histologically mimic non-alcoholic fatty liver disease. In this way, periodically re-evaluating these patients is vital for the perception of late-emerging diagnostic features, especially autoantibodies, metabolic variations, concurrent immune diseases, and liver biopsy findings.

Previously, Czaja had proposed a three-step algorithm to diagnose patients with cryptogenic chronic hepatitis, which has not been further validated in clinical practice (1). In this study, we aimed to investigate if patients with chronic hepatitis classified as cryptogenic by general practitioners may actually have a defined etiology if an appropriate diagnostic flowchart is followed, and to propose a new diagnostic approach incorporating metabolic-dysfunction-associated fatty liver disease (MAFLD) definition and LAL-D investigation.

## 2 Methods

### 2.1 Study population

The study population included adult (≥ 18 years old) patients diagnosed with cryptogenic chronic hepatitis by general practitioners and referred to four different Brazilian hepatology centers between 1 October 2016 and 30 November 2018 [Hospital das Clínicas of the University of São Paulo School of Medicine (HCFMUSP), Division of Gastroenterology (Gastrocentro) of the University of Campinas (UNICAMP), School of Medicine of Ribeirão Preto of the University of Sao Paulo (FMRP-USP), and Instituto Alfa de Gastroenterologia at Hospital das Clínicas of the Federal University of Minas Gerais (UFMG)]. All study procedures were conducted in accordance with the ethical standards of the Helsinki Declaration. The present study was approved by the Ethics Committee Boards of all institutions and individual informed consent was obtained from all participants at the time of blood collection for LAL-D enzyme activity measurement.

### 2.2 Data collection

Demographic, clinical, and laboratory data, as well as liver histology and imaging findings, were retrospectively collected from electronic or paper-based medical records. Liver cirrhosis was defined according to clinical, imaging, and histological findings. Alcohol intake was assessed (less than <140 g/week for both genders) to exclude alcoholic liver disease. The body mass index (body weight [kg]/body height [m^2^]) was calculated in all patients. Overweight and obesity were assumed if the body mass index exceeded 25 kg/m^2^ and 30 kg/m^2^, respectively. Metabolic parameters, including serum fasting glucose, triglycerides, and cholesterol, as well as blood pressure and waist circumference, were reviewed for MAFLD diagnosis (7). The diagnosis of MAFLD was based on evidence of hepatic steatosis [histological, imaging, or blood biomarker evidence (fatty liver index ≥60) of fat accumulation in the liver] in addition to one of the following criteria: overweight/obesity, presence of type 2 diabetes mellitus, or evidence of metabolic dysregulation. MAFLD-related cirrhosis patients are patients with cirrhosis, historical documentation of steatosis by hepatic imaging or biopsy, and past or present evidence of metabolic risk factors that meet the criteria to diagnose MAFLD. The diagnosis of non-alcoholic fatty liver disease (NAFLD) was established by the evidence of hepatic steatosis by either imaging or histology, and lack of secondary causes of hepatic fat accumulation (11). Wilson’s disease and alpha-1 antitrypsin deficiency were tested by determination of ceruloplasmin/urinary copper, and alpha-1 antitrypsin levels (or alpha-1 fraction of protein electrophoresis, in cases where there were not dosages of alpha-1 antitrypsin available), respectively. Females with transferrin saturation of >45% and serum ferritin of >200 μg/L and males with transferrin saturation of >50% and ferritin of >300 μg/L were submitted to genotyping for p.C282Y in HFE and screened for hemochromatosis. All patients were also assessed for serum markers of hepatitis B and C (anti-HCV, HBsAg, anti-HBc, HBV DNA, and HCV RNA by polymerase chain reaction), HIV infection, serum anti-nuclear antibody, smooth muscle antibody, anti-liver kidney microsomal antibody type 1, and anti-mitochondrial antibodies. The international autoimmune hepatitis score was calculated for patients with clinically suspected autoimmune hepatitis. Additionally, liver imaging tests (ultrasound, computer tomography, and magnetic resonance, when available) were evaluated for the exclusion of MAFLD and biliary and vascular etiology. Histopathology was assessed in patients who underwent liver biopsy or liver transplantation. Finally, Czaja’s algorithm for cryptogenic chronic hepatitis diagnosis was retrospectively tested to assess its performance in a real-life cohort of patients with presumed chronic cryptogenic hepatitis referred by general practitioners to specialized centers (1). Individuals with incomplete investigations were excluded from the database.

### 2.3 Lysosomal acid lipase enzyme activity

Lysosomal acid lipase (LAL) enzyme activity was measured in all patients to assess the prevalence of LAL-D among patients with cryptogenic hepatitis. All individuals had collected a blood sample of about 5 mL from peripheral venous access, which was then pipetted (four drops) on filter paper for preparing dried blood spots. The material was randomly referred for analysis at Seattle Children’s Hospital, USA, or AFIP (Research Incentive Fund Association), Brazil. LAL activity was measured by a highly specific test using lalistat 2, a specific LAL inhibitor at both laboratories. In Seattle, in-house methodology was considered. In the AFIP laboratory, the methodology described by Hamilton et al. was used (12). The dosage of LAL activity was generously provided by Alexion Pharmaceuticals (New Haven, Connecticut, USA).

### 2.4 Statistical analysis

A statistical analysis was performed using SPSS 25.0 software (IBM, USA). Continuous variables distribution was assessed using the Shapiro–Wilk test, and those with Gaussian distribution were expressed as mean and standard deviation, or as median and interquartile range (IQR) if there was a skewed distribution. Categorical variables were expressed as absolute number and percentage. Comparison between both algorithms presented were made by chi-square statistics, and a *p*-value < 0.05 was considered significant.

## 3 Results

**Figure 1:**
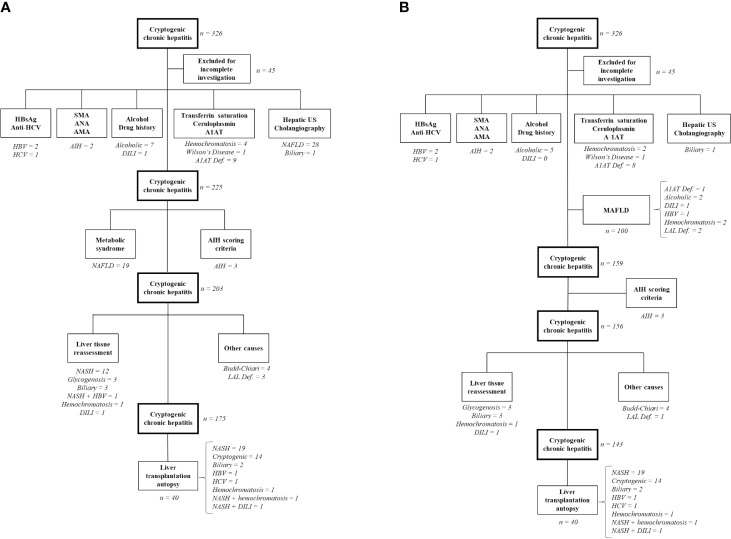
Algorithms for diagnosis of cryptogenic chronic hepatitis. **(A)** Original Czaja´s algorithm; **(B)** New proposed algorithm; After each etiology is presented the absolute number of patients diagnosed in each step. In brackets, it is described the number of patients with overlapping liver diseases and its respective etiologies. HCV, hepatitis C virus; HBV, hepatitis B virus; DILI, drug induced liver injury; US, ultrasound; A1AT, Alpha-1 antitrypsin deficiency; NAFLD, non-alcoholic fatty liver disease; NASH, non-alcoholic steatohepatitis; MAFLD, metabolic-dysfunction-associated fatty liver disease; AIH, autoimmune hepatitis; LAL def., lysosomal acid lipase deficiency.

Three hundred and twenty-six patients [mean age 60 (46–68) years, 42.9% male] were initially included, 35.7% with cirrhosis. Forty-five individuals were excluded due to incomplete etiological investigations. Clinical characteristics of the cohort are shown in **Supplementary Table 1**. Using Czaja’s algorithm, diagnosis of NAFLD could be established in 60 patients (21.3%), alpha-1-antitrypsin deficiency in nine (3.2%), alcoholic liver disease in seven (2.7%), autoimmune hepatitis in five (1.78%), hemochromatosis in five (1.78%), biliary-related hepatitis in four (1.4%), viral hepatitis in four (1.4%), Budd–Chiari syndrome in four (1.4%), glycogenosis in three (1%), drug-induced liver injury in two (0.7%), and Wilson’s disease in one (0.35%). In 158 patients, at least one liver biopsy had been performed. Etiological diagnosis was possible only by liver tissue evaluation in 21 individuals, most of them with NAFLD (57%). Finally, 175 out of 281 patients remained with cryptogenic hepatitis (62.3%). During follow-up, 40 of those patients were submitted for liver transplantation and 21 (52.5%) were retrospectively diagnosed with non-alcoholic steatohepatitis after a histopathological examination of the explanted liver (**Figure 1A**).

By including MAFLD at the first step of the new algorithm, 100 patients would have been diagnosed (35.6%), reducing the number of individuals without a diagnosis by 11.4%. On the other hand, nine (9%) of those patients presented with another concomitant liver disease diagnosis besides MAFLD (**Figure 1B**). LAL-D was demonstrated in three individuals (1%). Finally, using the new algorithm, 143 patients out of 281 (50.9%) would remain cryptogenic.

Comparing both algorithms, chronic liver disease could have an etiological diagnosis in 37.7% by Czaja’s algorithm and 49.1% by the new algorithm (*p* = 0.006).

## 4 Discussion

Accurate and timely diagnosis of a chronic hepatitis disease is fundamental to ensure patients receive appropriate treatment. Although there have been global improvements in analytical technology, developing nations such as Brazil face obstacles in enhancing their diagnostic abilities. These challenges stem from financial constraints, the concentration of resources in large urban centers, and a shortage of trained personnel (13). In our study, careful re-evaluation of all clinical, laboratory, imaging and histological data using Czaja’s algorithm could reduce the number of patients initially diagnosed with presumed cryptogenic hepatitis from 281 to 175 (–37.2%). Diagnostic yield could be improved by 11.4% by the inclusion of the new MALFD criteria and LAL-D investigation, although it is always a challenge to determine the best practical and feasible diagnostic strategy. The etiology of the liver disease was not defined in 50.9% of the cases using the new algorithm. Even in those who underwent liver transplantation, the histopathological examination of explanted liver could not define liver disease etiology in 35% of the cases.

The discovery of hepatitis B virus in the 1960s and hepatitis C virus in the 1990s have previously challenged the existence of cryptogenic chronic hepatitis (14, 15). Even after incorporating a viral hepatitis serological examination as part of the initial liver disease investigation, there can be still be cases of occult hepatitis C or B virus infection that can be perceived only after a direct liver tissue evaluation (16–18). In our study, two cases of occult viral hepatitis were observed in liver transplantation explants (5%), reinforcing the concept that viral hepatitis can still represent a small proportion of cryptogenic cirrhosis depending on the prevalence of the disease in the evaluated population. In fact, occult hepatitis C and B have been demonstrated in 8% (16) and 6.3% (18), respectively, of liver transplant patients with cryptogenic cirrhosis. Conversely, recent research indicates that the hepatitis B virus infection is responsible for a small percentage of cases (2%) of cryptogenic chronic hepatitis. Similarly, it is expected that hepatitis C virus infection also plays a minor role in this condition (19). Other viruses have also been investigated as etiological agents in the liver transplant population with cryptogenic cirrhosis, but with no definitive role, such as hepatitis G (20–22).

The obesity and metabolic syndrome epidemics in the last two decades have been dramatically associated with increasing occurrences of NAFLD. Interestingly, previous studies have shown that overweight and metabolic disorders are more likely to be found among cryptogenic chronic hepatitis subjects than in other causes of liver disease (8, 9, 23). Clark et al. have also demonstrated that patients with unexplained aminotransferase elevation present with significantly higher body mass index, waist circumference, triglycerides, and fasting insulin, and lower HDL—features associated with NAFLD (24). Furthermore, studies that evaluated histopathological findings in chronic cryptogenic hepatitis found a high frequency of fatty liver (28%–68%) (5, 25–28). Nevertheless, it is widely recognized that advanced hepatic fibrosis can alter, diminish, or even eliminate the histological characteristics of hepatic steatosis. This can result in the misidentification of individuals with NAFLD as having cryptogenic chronic liver disease. Previous studies have shown that cryptogenic cirrhosis presents more active fibrosis and a higher risk of liver-related clinical events than NAFLD-cirrhosis patients, highlighting the importance of differentiating both entities (29, 30). The new positive criteria for MAFLD minimizes this problem, especially for MAFLD-related cirrhosis, since histology is no longer required for diagnosis (7).

Histopathological examinations of the explanted liver after transplantation may significantly help to diagnose the cause of cirrhosis in patients with cryptogenic liver disease and guide specific post-transplantation therapies. NAFLD has been shown to be the most common cause of presumed cryptogenic liver disease diagnosed by histological evaluation of the explanted liver. In our cohort, non-alcoholic steatohepatitis was diagnosed in nearly 50% of the patients presenting for liver transplantation, which was similar to one other study evaluating liver explants (31). Conversely, Tardu et al. have shown a 25% rate of steatosis in the transplanted livers of patients with cryptogenic cirrhosis (6).

Our study has some limitations inherent to its retrospective design, such as some patients being lost due to missing data. A low incidence of autoimmune hepatitis in cryptogenic chronic hepatitis was observed, but it should be acknowledged that not all centers in Brazil have access to unconventional antibodies, such as anti-soluble liver antigen-liver/pancreas. Hepatic steatosis was detected by different methods—histological, imaging, or blood biomarker—which certainly present different accuracies. The high prevalence of cirrhotic patients may have been underestimated by the prevalence of fatty liver disease. Furthermore, liver biopsy was not performed in all patients. On the other hand, the large sample size and the multicenter data reflects real-life clinical practice. The implementation of the new algorithm would allow for an etiological diagnosis in 49.1% of the patients, reducing the necessity of tissue assessment in several patients.

## 5 Conclusions

In conclusion, a meticulous and consistent approach to patients with cryptogenic chronic hepatitis can result in an etiological diagnosis in almost 50% of cases. About one-third of patients initially diagnosed with cryptogenic liver disease were eventually found to have MAFLD. Although it is uncommon, testing for LAL-D should be considered for individuals with chronic liver disease of unknown origin. An updated diagnostic algorithm has been suggested for these individuals and should be tested in other groups to ensure its efficacy.

## Data availability statement

The original contributions presented in the study are included in the article/**Supplementary Material**. Further inquiries can be directed to the corresponding author.

## Ethics statement

The study was conducted in accordance with the Declaration of Helsinki, and approved by the following Institutional Review Boards [University of São Paulo School of Medicine (USP), University of Campinas (UNICAMP), Ribeirão Preto—University of Sao Paulo (FMRP-USP), and Federal University of Minas Gerais (UFMG)]. The patients/participants provided their written informed consent to participate in this study.

## Author contributions

GC, AC, DM, CO, and MG: study concept and design, acquisition and interpretation of data, and analysis drafting of the manuscript. PZ: acquisition of data and exam collection. M-CS, RG, RA, AA, CC, and MN: acquisition of data. AF and FC: analysis drafting. MN: statistical analysis. All authors read and approved the final manuscript.
