# Supplementary material for: Cryptogenic chronic hepatitis: looking for an ideal diagnostic algorithm
Source: Front Gastroenterol (Lausanne). 2023 Aug 1;2:1209000. doi: 10.3389/fgstr.2023.1209000 (PMC12952394; doi:10.3389/fgstr.2023.1209000)
Supplement: Supplementary file 1 [file Table_1.docx]

**Supplementary Table 1: Cohort characteristics**

| ***Variables*** | ***Overall Cohort (n = 326)*** |
| --- | --- |
| **Male sex** | 140 (42.9) |
| **Age (years)** | 60 (46-68) |
| **Cirrhosis** | 116 (35.7) |
| **Body mass index (Kg/m²)** | 27.6 (±5.9) |
| **Fasting glucose (mg/dL)** | 110 (±354) |
| **Triglycerides (mg/dL)** | 114 (±376) |
| **Cholesterol (mg/dL)** | 165 (±45.9) |
|  |  |
| **Excluded due to incomplete examination** | 45 (13.8) |
|  |  |
| ***Patients with complete examination*** | ***Analyzed Cohort (n = 281)*** |
| **Liver biopsy** | 158 (56.2) |
| **Etiological investigation by Czaja** |  |
| Cryptogenic hepatitis | 175 (62.3) |
| NAFLD | 60 (21.3) |
| *Concomitant hepatitis B virus* | 1 (0.3) |
| A1AT deficiency | 9 (3.2) |
| Alcoholic liver disease | 7 (2.7) |
| Autoimmune hepatitis | 5 (1.8) |
| Hemochromatosis | 5 (1.8) |
| Biliary-related hepatitis | 4 (1.4) |
| Budd-Chiari syndrome | 4 (1.4) |
| Viral hepatitis | 3 (1.0) |
| Glycogenosis | 3 (1.0) |
| LAL-D | 3 (1.0) |
| DILI | 2 (0.7) |
| Wilson’s disease | 1 (0.3) |
| **Etiological investigation by new algorithm** |  |
| Cryptogenic hepatitis | 143 (50.9) |
| MAFLD | 100 (35.6) |
| *Concomitant alcoholic liver disease* | 2 (0.7) |
| *Concomitant hemochromatosis* | 2 (0.7) |
| *Concomitant LAL-D* | 2 (0.7) |
| *Concomitant A1AT deficiency* | 1 (0.3) |
| *Concomitant DILI* | 1 (0.3) |
| *Concomitant hepatitis B virus* | 1 (0.3) |
| A1AT deficiency | 8 (2.8) |
| Alcoholic liver disease | 5 (1.8) |
| Autoimmune hepatitis | 5 (1.8) |
| Biliary-related hepatitis | 4 (1.4) |
| Budd-Chiari syndrome | 4 (1.4) |
| Hemochromatosis | 3 (1.0) |
| Viral hepatitis | 3 (1.0) |
| Glycogenosis | 3 (1.0) |
| LAL-D | 1 (0.3) |
| DILI | 1 (0.3) |
| Wilson’s disease | 1 (0.3) |
|  |  |
| ***Liver transplantation during follow-up*** | ***n = 40*** |
| **Explant histopathological evaluation** |  |
| NASH | 21 (52.5) |
| *Concomitant hemochromatosis* | 1 (2.5) |
| *Concomitant DILI* | 1 (2.5) |
| Cryptogenic hepatitis | 14 (35.0) |
| Biliary-related hepatitis | 2 (5.0) |
| Viral hepatitis | 2 (5.0) |
| Hemochromatosis | 1 (2.5) |

*Data are expressed as absolute number (percentage). A1AT, alpha-1-antitrypsin; DILI, drug-induced liver injury; LAL-D, liposomal acid lipase deficiency; MAFLD, metabolic-dysfunction-associated fatty liver disease; NAFLD, nonalcoholic fatty liver disease; NASH, nonalcoholic steatohepatitis.*
